# Microarray Data Standards: An Open Letter

**DOI:** 10.1289/ehp.112-1277123

**Published:** 2004-08

**Authors:** On behalf of the MGED Society, Catherine Ball, Alvis Brazma, Helen Causton, Steve Chervitz, Ron Edgar, Pascal Hingamp, John C. Matese, Helen Parkinson, John Quackenbush, Martin Ringwald, Susanna-Assunta Sansone, Gavin Sherlock, Paul Spellman, Christian Stoeckert, Yoshio Tateno, Ronald Taylor, Joseph White, Neil Winegarden

**Affiliations:** ^1^Stanford University, Stanford, CA, USA;; ^2^EMBL-The European Bioinformatics Institute, Cambridge, UK;; ^3^MRC Clinical Sciences Centre/Imperial College, London, UK;; ^4^Affymetrix, Inc., Emeryville, CA, USA;; ^5^The National Center for Biotechnology Information, Bethesda, MD, USA;; ^6^INSERM ERM 206, Marseille, France;; ^7^Carl Icahn Laboratory, Princeton University, Princeton, NJ, USA;; ^8^The Institute for Genomic Research, Rockville, MD, USA;; ^9^The Jackson Laboratory, Bar Harbor, ME, USA;; ^10^Lawrence Berkeley National Laboratory, Berkeley, CA, USA;; ^11^University of Pennsylvania, Philadelphia, PA, USA;; ^12^DNA Data Bank of Japan, Mishima, Shizuoka, Japan;; ^13^Pacific Northwest National Laboratory, Richland, WA, USA;; ^14^University Health Network, University of Toronto, Toronto, Ontario, Canada.

A fundamental principle guiding the publication of scientific results is that the data supporting any scholarly work must be made fully available to the research community in a form that allows the basic conclusions to be evaluated independently. In the context of molecular biology, this has typically meant that authors of papers describing a newly sequenced genome, gene, or protein must deposit the primary data into a permanent, public data repository such as the sequence databases maintained by the DNA Data Bank of Japan (DDBJ), the EMBL-European Bioinformatics Institute (EBI), and the National Center for Biotechnology Information (NCBI). Similarly, we, the members of the Microarray Gene Expression Data (MGED) Society (http://www.mged.org), believe that all scholarly scientific journals should now require the submission of microarray data to public repositories as part of the process of publication. While some journals have already made this a condition of acceptance, we feel that submission requirements should be applied consistently and that journals recognize ArrayExpress (Brazma et al. 2003), Gene Expression Omnibus (GEO; Edgar et al. 2002), and the Center for Information Biology gene Expression Database (CIBEX; Ikeo et al. 2003) as acceptable public repositories. To this end, the members of the MGED Society propose the following as a new paradigm for the publication of microarray-based studies:

1. Authors should continue to take primary responsibility for ensuring that all data collected and analyzed in their experiments adhere to the MIAME (Minimum Information About a Microarray Experiment; http://www.mged.org/Workgroups/MIAME/miame.html) guidelines and continue to use the MIAME checklist (http://www.mged.org/Workgroups/MIAME/miame_checklist.html) as a means of achieving this goal.
2. The scientific journals should require that all primary microarray data be submitted to one of the public repositories—ArrayExpress, GEO, or CIBEX—in a format that complies with the MIAME guidelines.
3. The public databases should work with authors and the scientific journals to establish data submission and release protocols to ensure compliance with MIAME.
4. To assist with the review process, the databases should continue to work in collaboration with publishers to provide qualified referees with secure means of access to prepublication data. Authors should be strongly encouraged to submit data to the databases during review.

Naturally, data should be protected from general release prior to either publication or authorization from the data submitters, whichever comes first. At a minimum, the journals should require valid accession numbers for microarray data as a requirement for publication, and these accession numbers should be included in the text of the manuscript to allow members of the community to find and access the underlying data.

Since its inception in 1999, the MGED Society has been working with the broader scientific community to establish standards for the exchange and annotation of microarray data. In December 2001, we proposed the MIAME guidelines (Brazma et al. 2001) and requested that interested parties provide feedback on its relevance and utility. The feedback from both researchers and scientific journals was overwhelmingly positive, yet almost everyone who responded also asked for help in implementing these guidelines.

Subsequently, in the summer of 2002, we submitted an open letter to various journals (e.g., Ball et al. 2002a, 2002b) urging the community to adopt the MIAME requirements for microarray data publication. We provided a checklist so that authors could ensure that sufficient information would be available to allow their data to be re-analyzed by others. Again, the response from the community was extremely positive, and most of the major scientific journals now require publications describing microarray experiments to comply with the MIAME standards. While the adoption of these standards has greatly improved the accessibility of microarray data, much of these data remain on individual authors’ websites in a variety of formats; consequently, obtaining and comparing data sets remains a significant challenge. Clearly we need additional requirements for publication that include submission of expression data to public data repositories.

Though one might ask why this requirement was not part of the original MIAME recommendation, the answer is quite simple—MIAME was ahead of its time. While the major public DNA sequence database groups at the NCBI and EMBL-EBI had developed nascent microarray data repositories, and work was under way to create a similar database at the DDBJ, submitting data to these databases was a considerable burden for authors. However, since that time, improvements in the data-entry utilities available for GEO (http://www.ncbi.nlm.nih.gov/geo), ArrayExpress (http://www.ebi.ac.uk/arrayexpress), and CIBEX (http://cibex.nig.ac.jp) databases, as well as a growing number of commercial and academic software packages capable of writing MAGE-ML documents (Spellman et al. 2002) that can be directly submitted to these public databases, have lowered the barriers for data submission to the point where we as a community must now reconsider that submission to one of these databases be a requirement.

Requiring authors to submit microarray data to the public databases will provide a number of distinct advantages to the entire research community:

- These established repositories have a commitment to continued community service and to providing some level of assurance that published gene expression data sets will continue to be available into the future.
- Having the data available in these public repositories in a standardized format will not only make it more accessible, but it will allow expression data to be integrated with other relevant data, including the available genome sequences, single nucleotide polymorphism (SNP) and haplotype mapping information, the literature, and other resources that can aid in further interpretation of expression patterns. Although many authors now provide some or all of this information, the established databases are much more likely to ensure that these links are maintained and current.
- Curation of data submitted to public data repositories will assist authors, reviewers, and publishers in ensuring that the data comply with the MIAME requirements, further enhancing its utility.
- The standardization of microarray data formats will enable the development of additional data analysis and integration tools and makes it easier for scientists to access, query, and share data.
- Finally, submission prior to publication will make it easier for referees to access the data confidentially, facilitating the review and publication process.

In the same way that availability of sequence data had a profound impact on a wide range of disciplines, we believe that requiring that microarray data be deposited into public repositories as a necessity for publication will accelerate the rate of scientific discovery.

What this proposal requires is a change in the way in which we approach the publication of microarray-based studies. Both authors and journals have a responsibility to ensure that the requisite data are available, and because submitting MIAME-compliant data can take considerable time and effort, this process should be factored into review and publication timelines. However, while this process may be time consuming and painful at first, we believe that the benefits of building an open repository of micro-array data will far outweigh any initial disadvantages. As always, it is our sincere hope that these suggestions stimulate discussion within the community and that together we can arrive at a consensus that ensures that microarray data are widely and easily accessible. Finally, we would like to urge the DDBJ, EMBL-EBI, and NCBI to work together toward exchanging all MIAME-compliant microarray data.
